# Phase I trial evaluating the antiviral agent Cidofovir in combination with chemoradiation in cervical cancer patients

**DOI:** 10.18632/oncotarget.8224

**Published:** 2016-03-21

**Authors:** Eric Deutsch, Christine Haie-Meder, Mohamed Amine Bayar, Michele Mondini, Mélanie Laporte, Renaud Mazeron, Julien Adam, Andrea Varga, Gilles Vassal, Nicolas Magné, Cyrus Chargari, Emilie Lanoy, Patricia Pautier, Antonin Levy, Jean-Charles Soria

**Affiliations:** ^1^ Department of Radiation Oncology, Gustave Roussy Cancer Campus, Paris-Sud University, Villejuif, France; ^2^ Drug Development Department (DITEP), Gustave Roussy Cancer Campus, Paris-Sud University, Villejuif, France; ^3^ Paris-Sud University, Kremlin-Bicêtre Medical University, DHU TORINO, SIRIC SOCRATES, LABEX LERMIT, Le Kremlin-Bicêtre, France; ^4^ I NSERM U1030 Molecular Radiotherapy, Gustave Roussy Cancer Campus, Villejuif, France; ^5^ Biostatistics and Epidemiology Unit, Gustave Roussy Cancer Campus, Villejuif, France; ^6^ Department of Medical Biology and Pathology, Translational Research Laboratory and Biobank (UMS3655 CNRS/US23 INSERM), INSERM Unit U981, Villejuif, France; ^7^ Department of Clinical Research, Gustave Roussy Cancer Campus, Paris-Sud University, Villejuif, France; ^8^ Department of Radiation Oncology, Institut de Cancérologie de la Loire-Lucien Neuwirth, Saint-Priest en Jarez, France; ^9^ Inserm U1018 Centre for Research in Epidemiology and Population Health, Paris-Sud University, Villejuif, France

**Keywords:** phase I, HPV, Cidofovir, radiotherapy, cervix cancer

## Abstract

**Purpose:**

This phase I trial aimed to assess the safety and determine the recommended Phase II dose (RP2D) of Cidofovir combined with chemoradiotherapy in patients with stage IB2-IVA cervical cancer.

**Experimental design:**

Incremental doses (1, 2.5, 5 and 6.5 mg/kg) of IV Cidofovir were administered weekly for two weeks, and then every 2 weeks from the start of chemoradiotherapy to the initiation of utero-vaginal brachytherapy. Biological expression of HPV was analyzed during treatment and tumor response was assessed according to RECIST v1.0 criteria.

**Results:**

A total of 15 patients were treated with Cidofovir. Dose-limiting toxicities occurred in 2/6 patients at the 6.5 mg/kg dose level (G3 proteinuria, and G3 acute pyelonephritis with G3 febrile neutropenia). No toxicity occurred at the 5 mg/kg dose level, but only 3 patients received this dose due to trial interruption because of low accrual. The most frequent G3-4 adverse effects observed during the trial were: abdominal pain (n=3), infection (n=2), leuckoneutropenia (n=2), and others (n=6). No toxic death or major renal side effect occurred. The best response was that 8/9 evaluable patients achieved a complete response (89%). In the intention to treat population, the 2-year overall and progression-free survival rates were 93% and 76%, respectively. Biological monitoring of HPV-related markers (decreased p16 expression, and increased p53 and pRb levels) was possible on sequential tumor biopsy samples. The genomic alterations identified were PIK3CA (n=5; one also had a KRAS mutation), and HRAS (n=1) mutations.

**Conclusion:**

Cidofovir at a dose of 5mg/kg combined with chemoradiotherapy appeared tolerable and yielded tumor regressions. Due to early trial interruption, the RP2D was not confirmed.

## INTRODUCTION

Platinum salt-based chemoradiotherapy (CRT) combined with brachytherapy is the standard treatment for locally advanced uterine cervix cancers (IB2 to IVa according to the International Federation of Gynecology and Obstetrics [FIGO] classification). In a meta-analysis, chemotherapy combined with radiotherapy led to a 6% improvement in 5-year survival and also reduced the risk of local and distant recurrences. Nevertheless, disease-free survival remains low in this setting. Women with locally advanced cervical cancer (stage IB2 to IVa) have a higher recurrence rate (58% at 5 years) and worse survival (80% for stage IB to 30% for stage III) than those with early-stage disease [1]. Research is therefore warranted to develop agents that may improve clinical outcomes.

During the past few years, Cidofovir, the antiviral agent used in preclinical models, has been shown to selectively radiosensitize cells infected with human papillomavirus (HPV) [2-5]. Over 90% of cervical carcinomas contain HPV DNA, especially serotypes hpv-16 and hpv-18 [6, 7]. The HPV genome encodes viral E6 and E7 genes that are implicated in tumorigenesis. In the host cells, E6 binds to p53 and induces an ubiquitin-mediated degradation process, while E7 destabilizes pRb promoting tumor carcinogenesis and cell cycle progression [8, 9]. Preclinical studies reported that the Cidofovir antiviral agent triggered antiproliferative activity against HPV-infected cells [10], resulting in downregulation of E6 and E7 oncoproteins at their transcriptional level, with subsequent reactivation of p53 and pRb expression [2]. This nucleosidic analog of deoxicidine monophosphate was found to be a radiosensitizer [2, 3], with anti-angiogenic [4] and antimetastatic effects [5]. We conducted a phase I trial to assess the safety and determine the recommended phase II dose (R2PD) of Cidofovir in patients with locally advanced cervical cancer receiving standard CRT and brachytherapy.

## RESULTS

### Patient baseline characteristics and exposure to treatments

Fifteen female patients were enrolled between July 2008 and March 2013. Patient baseline demographics and disease characteristics are shown in Table 1. The median age was 45 years (range, 28-61). Eleven (73%) patients had an Eastern Cooperative Oncology Group (ECOG) PS of 0, and stages IB2 (n=7; 47%) or II (n=6; 40%) disease at trial entry. No patient had para-aortic node involvement.

**Table 1:** Patient characteristics at inclusion

Characteristics	*N*=*15 (%)*
Median Age (years, range)	45 (28-61)
ECOG PS score	
0	11 (73)
1	4 (27)
Histology of primary tumor	
Squamous cell carcinoma	10 (67)
Adenocarcinoma	5 (33)
FIGO Stage	
IB2	7 (47)
II	6 (40)
III	1 (7)
IV	1 (7)

ECOG PS: Eastern Cooperative Oncology Group Performance Status; N: number of patients.

Overall, 7 (47%) patients received the initially planned dose of Cidofovir and all patients received more than 80% of the initially planned dose (at least 5 out of 6 injections). Five patients (33%) discontinued Cidofovir prematurely due to toxicity.

All patients completed both external radiotherapy (45 Gy in 25 fractions) and brachytherapy (15 Gy to the intermediate-risk CTV) without interruption, over a median duration of 52 days (range, 42-71).

Overall, 4 (27%) patients received the initially planned dose of concurrent chemotherapy with carboplatin and 15 (93%) received more than 80% (at least 5 out of 6 injections). Five patients (33%) discontinued carboplatin prematurely due to toxicity and one due to obstructive renal failure.

### DLTs, MTD, and safety profile

All patients were evaluable for DLT. An overview of Cidofovir dose levels and DLTs observed in treated patients is presented in Table 2.

**Table 2:** Overview of Cifdofovir dose levels and DLTs observed in treated patients

Dose (mg)	Treated patients (N)	Patients evaluable for DLT (N)	Occurrence of DLT (N)
1	3	3	0
2.5	3	3	0
5	3	3	0
6.5	6	6	2
**Total**	**15**	**15**	**2**

DLT: dose-limiting toxicity ; N: number of patients.

After starting with a Cidofovir dose of 1 mg per kilogram of body weight (mg/kg; n=3), the dose was increased to 2.5 mg/kg, and then to 5 mg/kg and no DLT was observed. The dose was then increased to 6.5 mg/kg and a first DLT was observed, namely G3 proteinuria. This dose level cohort was subsequently expanded to 6 patients, and a second DLT was observed among cohort subjects (G3 acute pyelonephritis with G3 febrile neutropenia). As a result of these DLTs in 2/6 patients at a Cidofovir dose of 6.5 mg/kg, further patient accrual was stopped at this dose level. Due to trial interruption because of the low recruitment, only 3 patients received the dose of 5 mg/kg, and no toxicity was observed at that dose level.

During the first 10 weeks of treatment, a total of 177 AEs were observed, and 13 were grade 3-4. The most frequent AEs (all grades) were: diarrhea (93%), nausea/vomiting (93%), abdominal pain (87%), asthenia (80%), and anorexia (67%) (Table 3). The most common G3-4 AEs were: abdominal pain (n=3), infection (n=2), leukoneutropenia (n=2), and others (n=6) (Table 4). There were 13 serious adverse events (SAE) in 6 patients. Among these 13 SAEs, 6 were grade 3-4, and 7 grade 1-2. No toxic death occurred.

**Table 3:** Most common AEs (≥20 % of patients overall) observed in the 15 patients during exposure to the Cidofovir and chemoradiotherapy combination (weeks 1 to 10)

AEs	Total	Related to treatment
	N (%*)	N (%*)
Nausea / vomiting	14 (93)	14 (93)
Diarrhea	14 (93)	14 (93)
Abdominal pain	13 (87)	2 (13)
Asthenia	12 (80)	7 (47)
Anorexia	10 (67)	8 (53)
Constipation	9 (60)	5 (33)
Urinary tract infection	7 (47)	4 (27)
Headache	7 (47)	1 (7)
Vaginal bleeding	6 (40)	0
Fever	6 (40)	2 (13)
Myalgia	5 (33)	1 (7)
Vaginal discharge	5 (33)	1 (7)
Pain in the lower limbs	4 (27)	1 (7)
Anxiety / depression	4 (27)	1 (7)
Dizziness	3 (20)	0
Weight loss	3 (20)	3 (20)
Hematuria	3 (20)	1 (7)
Dysesthesia	3 (20)	1 (7)

AEs: adverse events, N: number of patients, RT: radiotherapy.

* % of patients who experienced the adverse event / total number of patients

**Table 4:** Grade 3 or 4 AEs considered to be related to the Cidofovir and chemoradiotherapy combination (weeks 1 to 10) in the 15 patients

G3-4 AEs	Tota	Related to treatment
	*N (%)*	*N (%)*
Abdominal pain	3	2
Infection	2	1
Leukoneutropenia	2	2
Proteinuria	1	1
Myalgia	1	0
Vaginal bleeding	1	0
Asthenia	1	0
Insomnia	1	0
Hyperglycemia	1	0

AEs: adverse events, N: number of patients.

There was no treatment-related late (post-treatment) G3-4 toxicity. The late G3-4 toxicities observed were: abdominal pain (n=2), hot flushes (n=1), and anxiety/depression (n=1).

### Antitumor activity of Cidofovir and CRT

All patients had a tumor assessment at inclusion and during follow-up. The best response was 8/9 complete responses (89%) among evaluable patients. At the first evaluation (end of treatment), one patient experienced progressive disease.

In the intention to treat population, he median follow-up for OS was 46 months (95% CI: 24-59 months), 2-year and 4-year OS rates were estimated at 93% (95% CI: 70-99%) and 84% (95% CI: 56-96%), respectively. The median follow-up for PFS was 27 months (95% CI: 11-41 months). Two-year PFS was estimated at 76% (95% IC: 47-91). Four patients (27%) experienced progressive disease (local: n=2; regional: n=1; both regional and distant: n=1). Among the 4 patients who relapsed, 2 had adenocarcinomas and one had initial FIGO stage IV disease (Supplementary Table S1).

### Translational research

PCR analysis identified 10, and 3 patients with positive HPV16 (62.5%) and HPV18 (18.75%) genotypes, respectively. The remaining three patients were negative for both HPV16/18, and were therefore carriers of another high-risk HPV genotype. The analysis of E6 RNA expression by RT-qPCR yielded heterogeneous results and no consistent variation was observed (Supplementary Figure S1).

**Figure 1:**
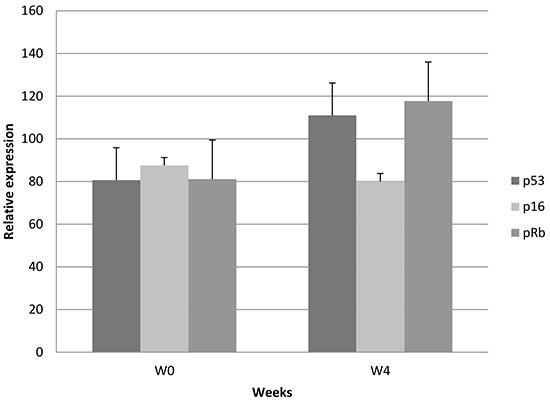
Relative IHC changes in p53, p16, and pRb

IHC analyses demonstrated a relative decrease in p16 (−9%) expression, and a relative increase in p53 (+37%; NS) and pRb (+45%; NS) levels at W4 compared with baseline (number of samples available for both time periods ≤ 7) (Figure 1).

IHC and PCR analyses were not possible at W10 because tumor cells were not present in the corresponding samples.

The genomic alterations identified were: PIK3CA (n=5; one also had a KRAS mutation), and HRAS (n=1) mutations. The other 8 genomic analyses available were negative. A PIK3CA mutation was found in one patient who experienced a local relapse (Supplementary Table S1).

## DISCUSSION

This is the first phase I trial to assess Cidofovir combined with CRT for previously untreated locally advanced cervical cancer. The R2PD was not confirmed due to premature trial discontinuation, but there was no DLT in patients who received Cidofovir at a dose of 5 mg/kg. Of note, the low accrual rate does not reflect a bias or excessive patient selection: it was due to the fact that DLTs were observed over 10 weeks and also because the trial acceptance rate was relatively low because of the sequential biopsies.

The predominant toxicity of Cidofovir is dose-dependent nephrotoxicity, including decreased renal function and the emergence of a Fanconi-type syndrome, with proteinuria. To prevent such effects, patients immediately received a saline hydration and probenecid before Cidofovir, which may prevent active renal tubular secretion and damage to proximal renal tubular epithelial cells [14]. Carboplatin was administered instead of cisplatin to further reduce potential cidofovir-related renal toxicity. Cidofovir is contraindicated in patients with significant proteinuria or chronic renal failure at baseline [15]. There was no major renal toxicity in this trial. One patient had G3 proteinuria, which subsequently resolved and there was one case of obstructive renal failure that may not be linked to the experimental agent. Further phase II studies evaluating the combination should include a safety run-in on the first patients, especially considering the small number of patients who received the dose of 5mg/kg in this study. Anyhow, the 5 mg/kg dose (per week for 2 weeks, then 5 mg/kg every other week) has yet to be validated as an effective and tolerable antiviral treatment [16].

Our results were encouraging in this patient population with locally advanced cervical cancer which also included adenocarcinoma histology [1]: the best response was 8 complete responses (89%), 4-year OS was 93%, and 2-year PFS was 76%. To the best of our knowledge, there is no other clinical experience with Cidofovir combined with radiotherapy. Cidofovir alone was also demonstrated to be active and well tolerated in the management of 89 patients with vulvar intraepithelial neoplasia [17]. The true impact of the combination of cidofovir and CRT on outcomes should be further assessed in a larger cohort of cervical cancer patients.

The combination of innovative agents and CRT for locally advanced cervical cancer should benefit from future biomarker-driven trials and new imaging modalities [18, 19]. It was suggested that IHC results may potentially serve as a prognostic tool and/or a surrogate marker of treatment response in advanced cervical cancers [20]. This was not the case (unselected population) in other early clinical trials combining the epidermal growth factor receptor (EGFR) inhibitor cetuximab with CRT in cervical cancers [21, 22]. IHC analysis of the samples collected here demonstrated a relative decrease in p16 expression, and a relative increase in p53 and pRb expression (Figure 1). On the other hand, no subsequent decrease in the E6 oncoprotein was observed. Tissue heterogeneity, arising from different proportions of tumor and healthy tissue, and the small number of samples analyzed may explain why no differences were observed. Moreover, we could not perform the analysis at W10 because no residual tumor tissue was found in those samples. These results should therefore be confirmed in a larger series of patients.

During the last decade, somatic mutations such as PIK3CA, PTEN, TP53, STK11, KRAS, and others have been described in the pathogenesis of cervical carcinomas [23]. PIK3CA is one of the most frequent (22-31%) mutations and is correlated with poorer outcomes and responses following CRT [22, 24, 25]. In our experience, there were 5/14 (36%) PIK3CA mutations and only one patient with this genomic alteration developed a local relapse.

Some of the effects of ionizing radiation are now recognized as contributing to antitumor immunity [26, 27]. Our group recently reported the combination of irradiation and a HPV vaccine as an effective anticancer treatment. The combined treatment additionally induced high levels of tumor-infiltrating, antigen-specific CD8(+) T cells, and CD8(+) T-cell memory, that are implicated in the antitumor activity [28]. Targeting molecules that downregulate the T cell immune response with immunotherapy such as anti-CTLA-4 (cytotoxic T-lymphocyte antigen-4) or anti-PD-1/PD-L1 (programmed death-1 and it ligands) is currently being assessed in patients with recurrent/metastatic cervical cancer (NCT01693783; NCT02257528; NCT01975831).

Finally, antiviral strategies remain relevant given the burden of HPV-related cervix and head and neck cancers in developed western countries. Cidofovir has also demonstrated antitumor activity in other malignancies such as glioblastomas [29]. Improved pharmacological versions (including nanotechnology) of cidofovir are becoming available and will increase the efficacy/tolerance ratio [30, 31]. Another phase I/II with such antiviral compounds will shortly be initiated in our institution for patients with locally advanced cervical cancer.

## PATIENTS AND METHODS

### Patients

Patients, aged between 18 and 70 years, were eligible for inclusion if they had histologically confirmed squamous cell carcinoma or adenocarcinoma FIGO stage IB2 (> 4 cm), II, III or IVA, regardless of the pelvic lymph node status (surgical exploration was optional), but without para-aortic metastasis. Additional inclusion criteria were: detection of the HPV genome in the initial tumor (Hybrid Capture II assay; Roche, France); life expectancy > 3 months; a negative β HCG test for premenopausal women; Eastern Cooperative Oncology Group Performance Status (ECOG PS) 0 or 1 at trial entry; adequate hematologic, hepatic, and renal functions. Exclusion criteria comprised: a history of cancer other than basal cell carcinoma; prior radiotherapy or chemotherapy; pregnancy; prior or current psychiatric illness; active infection or another serious underlying pathology that could prevent the patient from receiving the treatment (especially liver, nephrological, or heart conditions); inclusion in another clinical trial with an experimental compound (during this study or one month prior to enrollment); inability to adhere to study follow-up requirements for geographical; social or psychological reasons.

### Study design and treatments

This phase I study was an academic single-center, open-label, dose-escalation trial using a 3+3 design to evaluate the safety of Cidofovir combined with platinum salt-based CRT followed by brachytherapy in locally advanced cervical cancers. Intravenous (IV) Cidofovir was administered weekly, over two consecutive weeks (over 1h, and preceded by 1.5 liter of normal saline and oral probenecid, 2g 3h prior and 1g 2h and 8h following Cidofovir) and then every two weeks from the start of CRT (45 Gy delivered to the pelvis and IV weekly carboplatin AUC 2.5; carboplatin was administered instead of cisplatin due to potential renal Cidofovir-related toxicities) until the initiation of utero-vaginal brachytherapy (15 Gy equivalent dose delivered to the intermediate-risk clinical tumor volume [CTV]) up to a total of 6 injections in 10 weeks. Additional external radiotherapy was delivered (lymph node boost) no more than 3 days after brachytherapy if necessary. Incremental doses (1, 2.5, 5, and 6.5 mg per kilogram of body weight) of Cidofovir were administered in sequential cohorts of three patients. If no DLT was observed, the dose was escalated for the next cohort of three patients to the next higher dose level. If two or three DLTs were observed then the dose was de-escalated to the prior dose level. If one DLT was observed, three additional patients were treated at the same level. Afterwards, the dose was escalated only if no additional DLT was observed. The maximum tolerated dose (MTD) was the highest dose level at which less than two out of six patients experienced a DLT. All dose escalations were started once all patients in the previous cohort had completed the treatment. Patients had to be replaced if they were not fully evaluable for these assessments. The study was approved by the relevant ethics committee/institutional review board and was conducted in compliance with the Declaration of Helsinki and good clinical practice guidelines. Written informed consent was obtained from all patients before trial initiation.

### Objectives and outcome measures

The primary objective was to assess the safety of Cidofovir given concurrently with radiotherapy and to determine the MTD of Cidofovir. The secondary objectives were to collect data regarding antitumor activity of the combination and to evaluate the biological impact of the treatment (cf. next chapter).

DLT was assessed during treatment until week 10 according to Common Terminology Criteria for Adverse Events (CTCAE) v3.0, and was defined as any of the following adverse events (AEs) : grade (G) 4 neutropenia lasting more than 7 days, febrile neutropenia (> 38.5°C), G4 thrombocytopenia, G3-4 infection, G4 elevated serum creatinine, G3 proteinuria, G3-4 cardiac, neurological, liver or auditory toxicity, G4 digestive toxicity, interruption of radiotherapy > 7 days or a delay between radiotherapy-brachytherapy > 15 days due to toxicity, persistence of G3-4 hematological or renal toxicity > 21 days after the end of treatments, less than two injections of Cidofovir during CRT, and a toxicity-related death. The investigators had to evaluate any potential relationship between reported AEs and the experimental treatments. If no alternative cause of toxicity during treatment was identified, the observed AE had to be considered as related to the study drug. Antitumor activity was determined 6 to 8 weeks after treatment completion based on computed tomography (CT) and magnetic resonance imaging (MRI) according to Response Evaluation Criteria In Solid Tumors (RECIST) version 1.0.

### Translational research

HPV oncoprotein expression (E6, p53, and pRb) was analyzed during treatment based on sequential cervical biopsies performed at baseline, week [W]4, and W10. Formalin-fixed, paraffin-embedded material from each case was evaluated for p53/p16/pRb expression using immunohistochemistry (IHC). HPV 16/18 DNA and E6/E7 messenger RNA (mRNA) transcription using polymerase chain reaction (PCR) and *in situ* hybridization (via Life Technology) were used as previously described [11, 12], respectively.

Genomic analysis was performed on accessible samples. The mutational status of specific cancer genes was determined using next generation sequencing based on the Ion Torrent approach with the Ampliseq Cancer Hotspot panel V2 (Life Technologies, Darmstadt, Germany) library preparation, as previously described [13].

### Statistics

Progression-free survival (PFS) was defined as the time in months from the date of Cidofovir initiation to the date of progression or death from any cause or the date of the last evaluation for progression. Overall survival (OS) was defined as the time in months from the date of Cidofovir initiation to the date of death from any cause or the date of the last news. The patients’ vital status was systematically updated at the end of 2014. PFS and OS were estimated using the Kaplan-Meier product limit estimator. The p values of the log-rank test and 95% Rothman's confidence intervals are provided. The median follow-up time (months) was computed by the reverse Kaplan-Meier method. Analyses were performed using SAS Software, version 9.3 (SAS Institute, Cary, NC).

## CONCLUSION

In this phase I trial, Cidofovir (5 mg/week) combined with platinum salt-based CRT in previously untreated locally advanced cervical cancer appeared well tolerated. The RP2D could not be confirmed due to premature trial discontinuation. Further investigation is needed to better assess the therapeutic ratio of this new combination in trials that take into account modern radiation delivery techniques, and incorporate the emerging information on tumor biology.

## SUPPLEMENTARY FIGURE AND TABLE


