# Supplementary material for: Phase I trial evaluating the antiviral agent Cidofovir in combination with chemoradiation in cervical cancer patients
Source: Oncotarget. 2016 Mar 21;7(18):25549–57. doi: 10.18632/oncotarget.8224 (PMC5041925; doi:10.18632/oncotarget.8224)
Supplement: Supplementary file 1 [file oncotarget-07-25549-s001.pdf]

SUPPLEMENTARY FIGURE AND TABLE

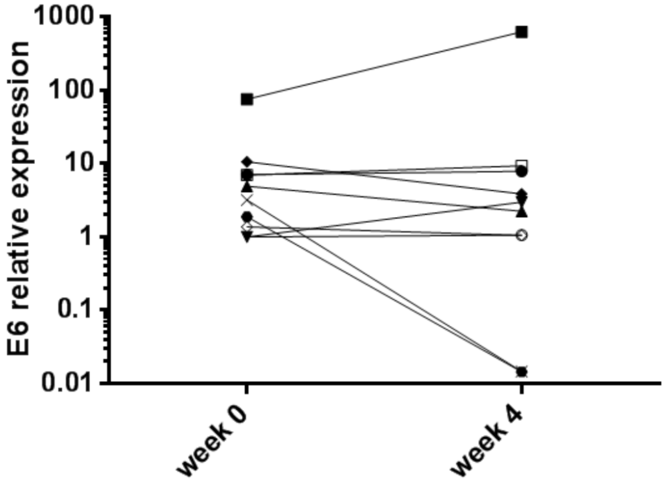

Supplementary Figure S1: E6 expression levels are heterogeneous among patients. The levels of E6 transcript of HPV16 (filled symbols) or HPV18(empty symbols) were evaluated by RT-qPCR performed on RNA extracted from tumor biopsies before the beginning of treatment (week 0) and after cidofovir administration (week 4).

Supplementary Table S1: Pattern of relapse according to clinico-biological characteristics

patient #	FIGO stage	Histology	Genomic mutation	Pattern of relapse
5	IIA	SCC	PI3KCA	L
8	IB2	ADK	WT	R+D
9	IB2	ADK	WT	R
12	IVA	SCC	WT	L

SCC: Squamous cell carcinoma; ADK: Adenocarcinoma; WT: wild type; L: local; R: regional; D: distant.
